# Serious game in developing emotional competence: An evaluation report

**DOI:** 10.12688/openreseurope.21634.2

**Published:** 2026-04-09

**Authors:** Maria Cassar, Daren Chircop, roderick Bugeja

**Affiliations:** 1 Department of Nursing, University of Malta, Msida, Tal qroqq, MSD0208, Malta

**Keywords:** Serious Game; Nurse education; emotional competence

## Abstract

**Background:**

The importance of the development of emotional competence amongst health care professionals is widely emphasised in the literature. The use of serious games in this regard is to-date in its infancy.

**Methods:**

This paper reports a evaluation exercise which was carried out to evaluate the development and application of a Serious Game as an educational resource amongst nursing students. The educational purpose of the game was to enable and support the development of emotional competence amongst nursing students. Undergraduate nursing students were invited to play the game and to participate in two focus group discussions which sought to evaluate the game as an educational tool. Eight nursing students participated in the evaluation exercise. Students’ experience and feedback were collected via two audio-recorded, transcribed focus group discussions lasting about an hour, with participant consent. Guided by Braun and Clarke’s guiding steps for data analysis, entailed the thematic analysis of the transcribed focus group discussions was carried out by the authors.

**Results:**

Three themes were derived from the data analysis; Perceived motivation for participation, Positive aspects of the workshop using Serious Game, and Future Initiatives.

**Conclusions:**

The use of serious games as an educational tool for developing emotional competence holds potential which merits further study and investigation. Targeting educators and game designers, the findings of the evaluation exercise are discussed and recommendations for future similar initiatives are shared in view of contributing to the development of similar educational initiatives.

## Plan language summary

The funded project included the design of a Serios Game for nursing students to use in seeking to build emotional competence. Emotional competence is a very important element of nurse practice. The University of Malta was a partner in the project and contributed towards the design and testing of the developed game. The university of Malta engaged in a formal evaluation exercise of the use of the developed game as part of a wider initiative, a workshop, for nursing students regarding emotional competence. This paper reports this evaluation exercise in view of sharing lessons learned and suggesting recommendations for parties planning similar initiatives.

## Introduction

Emotional competence is defined as the functional capacity wherein a human can reach their goals after an emotion-eliciting encounter ( Saani, 1999). The literature reports consensus about the importance of emotional competence in the performance of all professionals ( Ikävalko *et al*., 2020; Stamouli & Gerbeth, 2021). The importance of emotional competence is further accentuated in the practice of healthcare professionals since their practice entails direct interaction between humans ( Giménez-Espert *et al*., 2023; Jiménez-Picón *et al*., 2021). Moreover, the prevalence of disparities, including levels of knowledge and skills, across the various human players involved in the practice of a profession underlines further the importance of emotional competence in professional practice, especially in health and social care contexts ( Ikävalko *et al*., 2020). Emotional competence is associated with increased ability to bridge or level out such disparities ( Jiménez-Picón *et al*., 2021) and to dampen the unfavourable effect and impact that such disparities may have on professional relationships and their outcomes ( Ikävalko *et al*., 2020).

This paper argues that nursing students must develop emotional competence during their training, as it is essential for effective care. The emotional labour in nursing, ( Hochschild, 1983) has long been recognised and the building capacity to address emotional has been increasingly valued as a cornerstone of nurse education in view of enabling nurses to manage the psychological demands of healthcare ( Costa *et al.*, 2025). In nursing, emotional labour is the act of managing feelings and emotional expressions to fulfill the professional role ( Staden, 1998). Therefore, pre-registration education should provide appropriate opportunities to build this competence ( Giménez-Espert *et al*., 2023; Horton-Deutsch & Sherwood, 2008). Notwithstanding the above, nurse educators and nursing students alike acknowledge the challenges associated with the effective and efficient development of emotional competence ( Di Lorenzo *et al*., 2019) including time constraints, resources limitations, and ethical issues. There is a school of thought which argues that nursing students’ learning has been more focussed on developing clinical skills, leaving them poorly trained to deal with emotional situations ( Giménez-Espert *et al*., 2023). Another school of thought which stems from the observed increasing attention to environmental and planetary care in nurse education programmes ( Asaduzzaman *et al*., 2022; Vandenberg, 2023) shares concerns about further alienation of emotional development nurturing in pre-registration nurse education. The persistent increase in the inclusion of technology in care delivery is also frequently identified as a serious distraction, indeed a threat, to the development and practice of emotional competence amongst students and qualified staff ( Jawabreh, 2024; Robichaux *et al*., 2019; Waight & Holley, 2020; Zuzelo *et al*., 2008).

Current evidence ( Taylor *et al*., 2023) shows that the effectiveness of initiatives depends on the specific respective context where an initiative is delivered. Therefore, new teaching and learning methods should be developed and evaluated based on the context in which they are implemented ( Adami & Kiger, 2005; Asaduzzaman *et al*., 2022; Cubit & Ryan, 2011). This paper provides a detailed account of implementing and utilising an innovative Serious Game designed to foster emotional competence among student nurses enrolled on bachelor’s pre-registration nurse education programmes within the European Union (EU). As opposed to entertaining games, serious games are designed for an educational purpose ( Nazry *et al.*, 2017) and are typically entertaining tools with a purpose of education whereby, through gaming, players gain knowledge and practise skills through overcoming numerous hindrances in the Serious Game. The development of this Serious Game was a principal output of an EU-funded project, SG4NS, supported under the ERASMUS+ programme by the European Commission. The educational context involves a pre-registration curriculum comprising 4,600 hours of teaching and learning, with at least 2,300 hours dedicated to practical learning experiences and no less than one-third allocated to theoretical instruction. The Serious Game was specifically created to offer students an immersive and safe environment that supports robust experiential learning for a diverse group of learners. It served as a core educational tool during a workshop on emotional competence. This paper reports on the evaluation of the workshop, aiming to assess the Serious Game’s effectiveness as a learning resource.

The use of Serious Games in nursing education is well-documented, with studies suggesting that technology can enhance nursing students’ content knowledge ( Goodchild, 2018; Harerimana & Mtshali, 2020). While technology’s broader impact has been widely researched, findings remain contested. Notably, there is limited research on how Serious Games may influence the development of soft skills like emotional competence ( Saab *et al*., 2021; Sanchez-Valdeon *et al*., 2024). This paper aims to address that gap.

## The evaluation exercise

### The Serious Game

The Serious Game, developed by European universities from Portugal, Italy, Malta, Romania and Spain through the ERASMUS+ SG4NS project, seeks to help nursing students build emotional skills. Using an escape room format, it evokes emotions like curiosity and fear with challenges such as cockroach-infested bins and dark spaces. Players solve puzzles for at least 15 minutes in VR, then discuss their experience with a nursing educator.

### The workshop

A 3-day emotional competence workshop, led by two trained nursing educators, included presentations, videos, reflections, group activities, and assessments using PANAS ( Watson *et al*., 1988) and EVCE-r33 ( Veiga-Branco, 2021). It aimed to help students recognize, understand, and manage their own and others’ emotions constructively. Key topics included handling emotions in healthcare, supporting patients and relatives, empathy, assertiveness, and teamwork. Evaluation was conducted through structured focus groups.

The game provided students with a safe, immersive environment to experience and process emotions, guided by workshop moderators from the Department of Nursing at the University of X. Unlike real-life contexts that are harder to control, the Serious Game facilitates emotion elicitation and reflection in a secure setting, ensuring participants’ safety throughout. This controlled environment is particularly valuable for emotional learning and processing.

### Purpose

The purpose of the evaluation was to gain insight into the experiences and perspectives of the participants of the serious game as an educational tool.

## Methods

### Design

The evaluation exercise employed seeking feedback from the participating students through asking them a series of questions through in-person focus group discussions. As this exercise was specifically an evaluation of an educational initiative rather than a formal research study, institutional ethical approval was not required at this stage. However, it should be noted that should similar work be conducted in the future for research purposes, obtaining ethical clearance from the appropriate institutional review board will be mandatory to ensure the protection of participants and uphold research integrity. Permission to conduct the workshop and its evaluation was granted by the university management. Institutional ethical clearance was not required because the evaluation exercise reported in this paper was conducted as an educational evaluation exercise specifically designed to evaluate the implementation of a new pedagogical tool within the Department of Nursing. The exercise focused exclusively on collecting students’ perspectives in view of evaluating the new Serious Game rather than generating research data. Nevertheless, drawing upon the University of XXX Research Code of Practice and Research Ethics Review Procedure, applicable safeguards were implemented to ensure the informed participation of respondents, to prevent coercion, and to protect the confidentiality of participants including the use of an intermediary for recruitment, the submission of formal informed consent by participants and the use of pseudonyms.

### Setting and recruitment

Participation was open to undergraduate nursing students registeredthe University of Malta at the time, with no discrimination based on age, nationality, race, or gender. Undergraduate nursing students span three years of a Bachelors degree programme in nursing studies. An intermediary invited all undergraduate nursing students registered at the University of Malta via email, outlining the workshop’s focus on using VR to develop emotional competence in healthcare, session details, moderator info, and registration instructions. A certificate of attendance was offered. Consent to participate in the evaluation was given through a written email to University management. Eight students reported their interest to participate in the focus groups by the stipulated deadline. These students attended the workshop as one group. All completed the workshop in its entirety. They were randomly distributed evenly across the two focus groups discussions after the completion of the workshop.

### Data collection

Data were collected via two audio-recorded, transcribed focus group discussions lasting about an hour, with participant consent. The discussion focused on: (1) factors facilitating the workshop including number of participants and venue; (2) implementation challenges; (3) perceived achievement of outcomes; and (4) recommendations for future emotional competence interventions. The discussions also covered learning content, resources, space, duration, structure, and nursing educators’ performance. One author created the focus group’s discussion guide, which was reviewed by the other authors Two doctoral qualified senior academics moderated the sessions. Afterwards, one author analysed the data and developed themes, which all authors then reviewed and finalised together.

### Data analysis

The analysis of the recordings of the focus groups discussions was guided by thethe six steps of thematic analysis ( Braun & Clarke, 2006) noted in the table below ( Table 1). Organising the process of analysis through such set of steps provided opportunity for structure and rigour; required elements of valid evaluation. One author conducted the analysis of the focus groups discussion following the six steps. The others audited the process and regular debriefing meetings between the three were held at the end of each of the six steps of the process. After reading the transcripts the coding of all the transcripts ensued. Once all the content of the transcripts was coded, the emergence of themes from different sets of codes was explored and the names of themes were considered until an agreement was reached across all authors. During such meetings potential biases during the data collection and analysis were checked and any diverging interpretations across the authors of the students’ responses were resolved through revisiting students responses together and revised consideration and discussion of interpretation, until consensus amongst all authors was reached. Excerpts are provided to support theme development, allowing readers to trace authors’ interpretations. The selection of excerpts was also discussed across the authors.

**Table 1.** Six steps of thematic analysis ( Braun & Clarke, 2006).

Step	Description
1	Reading and re-reading the transcripts to familiarise oneself with the data
2	Coding the data
3	Examining the codes to generate initial themes
4	Reviewing these themes
5	Developing a detailed analysis of each theme and naming the themes
6	Writing a report which is presented below

## Findings and discussion

All participants agreed the workshop was valuable. Their feedback can be summarised in three main themes. Participant excerpts in the following sections comprise ellipses (...) which indicate omitted text for clarity ( Table 2).

**Table 2.** Participant excerpts in the following sections comprise ellipses (...) which indicate omitted text for clarity.

Theme	Description
Perceived motivation for participation	Reasons for joining the workshop
Positive aspects of the workshop using Serious Game	Benefits and strengths of the workshop
Future Initiatives	Suggestions for future emotional competence training

### Perceived motivation for participation

Most participants attended the workshop to learn how VR could help nurses develop emotional competence in clinical settings. They were keen to see how this technology might improve patient interactions and their own emotional management.

The focus on VR and emotional skills attracted them as a practical and innovative learning opportunity for nursing practice.

“ *I never went to a workshop before but, because it involved virtual reality and emotional competence, I was really interested in how these two are connected.*” (Participant 3).

“ *I was really interested in how virtual reality is associated with emotions, and how it can help us, as nurses, work in the clinical setting.*” (Participant 4).

Some participants joined the workshop to support their professional development, recognizing emotional competence as essential to nursing. They highlighted its impact on patient care and aimed to improve their ability to manage their own emotions and respond empathetically to others.

“ *For me, it was purely personal development. It was not just virtual reality.*” (Participant 2).

“ *Knowing it is about emotional competence … I know how important it is to nursing.*” (Participant 8).

The participants highlighted that emotional competence is important to the nursing profession because, in the clinical setting, nurses are bombarded with a range of emotions as they care for patients who are in different stages of their illness trajectory and hence, are experiencing different emotions. This suggests that nurses need to be aware of how to tackle such emotions.

“ *During placements, emotions are continuous … During placements, I tend to be overwhelmed with emotions.*” (Participant 3).

*“During placements, you care for one patient, and you experience certain emotions. Then you go next to another patient, and you feel other emotions. Emotions can be … opposite with the next patient. There are extremes.*” (Participant 5).

Other participants described how emotional competence is not only important when caring for patients but is also important to understand and manage emotions when around other nursing staff. They highlighted its importance in fostering a positive work environment, resolving conflicts, and supporting team cohesion. The responses of the participants suggest that emotional competence is seen as essential for both patient care and professional relationships with other nurses.

“ *During my placements, I struggle more with my colleagues than with the job itself. Hence, having emotional competence in relation to my colleagues, it really helps.*” (Participant 3).

“ *During my placements, I am so anxious. I am not anxious about the patients, but whether I am going to be accepted as part of the nursing team.*” (Participant 4).

One of the participants also stated that the way the workshop was advertised made it more intriguing and hyped her expectations. This participant appreciated the fact that the advertisement was rather vague and did not provide details about the particular topics to be covered over the 3 days. Instead, the advertisement simply stated that the workshop will comprise the use of VR in developing emotional competence in healthcare professionals, whilst also providing details regarding the duration and location.

“ *Being kind of vague about virtual reality and emotional competence made it more intriguing and increased my interest in applying. Providing more information about which topics we will be discussing could have led to us thinking that we would have already covered it in class … we would not have attended.*” (Participant 5).

### Positives aspects of the workshop using Serious Game

By the end of the workshop, participants felt more comfortable identifying and managing their emotions and better understood others’ emotions. The responses of the participants suggest that they found the training enhanced their emotional competence and believed this confidence would benefit both their professional and personal lives.

“ *Now we are more confident and comfortable in sharing our emotions when compared to the first day.*” (Participant 1).

“ *Now, I am more confident in understanding what the other person might be feeling.*” (Participant 4).

The approach taken during this workshop was an important factor which led to the participants feeling more confident in emotional competence at the end of the workshop. The responses of participants suggest that the fact that they were not pressured to share and were allowed to choose in which groups to discuss the various exercises was regarded favourably; it helped them to become more confident in expressing their emotions, not just with the people they knew, but also with others whom they did not know so well.

“ *Being put in a group which we do not know will be a problem. Once you open up with people you know then, it becomes easier to open up with others, whom you may not know so well.*” (Participant 4).

The number of participants who participated in the workshop was another important factor. All participants agreed that 10 students was the ideal number and it is imperative that the same participants continue to participate in such workshops, as highlighted in the excerpt below.

“ *Being in a small group made it easier for us to talk to the others, to get to know the others and to express our emotions … I think 10 students was a good number.*” (Participant 1).

“ *The fact that the same number of people remained throughout the whole workshop. If other people had joined later on then it would make it more difficult to open up because I would not have become comfortable with the new people who join.*” (Participant 4).

Another factor which made it easier for the participants to express themselves was the fact that the participants were all coming from the nursing profession and hence, they could understand each other because they would have experienced similar experiences in the clinical setting. This common ground appears to have fostered a sense of trust, as they had likely faced similar challenges and situations in the clinical setting. The participants’ responses suggest that this allowed the participants to communicate more openly and effectively, as they did not need to explain the nuances of their work or the context of their experiences, leading to richer and more meaningful discussions.

“ *The fact that we are all nurses. If we had people from other courses then it would have been difficult to express ourselves because they would not know what we are going through.*” (Participant 7).

The room size was ideal, offering comfort through adequate personal space and privacy through closed doors, that encouraged open discussion.

“ *The size of the room is important. If you are a small group, you need a small room to give more intimacy … the size of this room was perfect.*” (Participant 4).

“ *If we had met in the auditorium, which is a much bigger room, it would have been more difficult to share our experiences.*”

The participants were also satisfied with the content of the workshop. Videos, questionnaires, and PowerPoint presentations were all highlighted as playing an important role in helping the participants to think about their emotions.

“ *The videos. Watching the video for the second time and analysing it really helped. It is interactive. It is engaging.*” (Participant 5).

“ *The questionnaire. Learning something new about myself. Those kept me intrigued.*” (Participant 4).

Participants agreed the Serious Game effectively evoked a range of emotions, such as fear and satisfaction. They cited specific in-game experiences—like whispers inducing fear that influenced decision-making—to illustrate the game’s immersive and emotionally engaging nature. Post-game discussions seem to have enabled reflection, deepening their understanding of emotional competence.

“ *The virtual reality made it easier to understand how to handle certain emotions. And how emotions impact your actions. I was thinking about the game and made it easier to relate to.*” (Participant 1).

“ *The game really helped in the workshop … it provided a lot of examples for discussion. Most of us were able to relate because most of us felt the same emotions during the game.*”

### Future initiatives

Participants agreed that workshop presentations should be limited to one hour. When a presentation exceeded two hours on the third day, many found it hard to focus beyond the first hour. They recommended keeping presentations shorter and more focused to improve learning and participation.

“ *Maybe shorter presentations, such as one hour long. Long presentations make it difficult to concentrate.*” (Participant 4).

One of the participants highlighted the importance of providing short breaks throughout the workshop. This can help the participants to release some of the emotions felt, for example whilst watching a powerful video or participating in a hot discussion.

“ *Give short breaks throughout the workshop. To refresh. To help people release some of the emotions felt.*” (Participant 2).

The participants also highlighted the importance of focussing on specific topics which were touched on during the workshop but, which in their opinion, should be given more time and importance when discussing emotional competence. Such topics include assertiveness and conflict resolution.

“ *Conflict resolution. This workshop gave me an idea of what to do but still more information would be helpful.*” (Participant 3).

“ *Even confrontation. Finding the guts to speak up. I know I have to be more assertive and speak up, but I need the guts to speak up.*” (Participant 4).

### Implications and recommendations

Based on their reflections, the nursing educators recommend limiting the gaming exercise to 10 minutes, as longer durations may hinder learning. Extended play was observed to cause distraction and reduce engagement with the game’s educational purpose. The escape room design, comprising a mystery solving exploratory nature, potentially compromises the learners’ focus on emotional observations because the resolve to find a solution to the mystery may overwhelm other emotions. This risk is believed to increase with time duration of game playing. Educators also recommend offering brief VR headset tutorials for new users, with extra time to address any learning gaps. Indicated changes to the escape room design are found in the Table 3 below.

**Table 3.** Indicated changes to the escape room design are found in the Table 3.

Recommendation	Details
Clearly defined sequential areas	May be considered
Varied emotions	Inclusion recommended
In-game characters	Incorporation to be factored in

Alongside suggestions regarding the Serious Game, the authors also provide recommendations for nurse educators on supporting the development of emotional competence in nursing students ( Table 4).

**Table 4.** Recommendation & Main Focus.

Recommendation & Main Focus	Key Points
1. Universal nursing students’ emotional competence requirements	Discuss requirements at European and global levels; universal agreement needed; guide future research; design universally applicable interventions.
2. International nurse educators’ educational opportunities	Develop and implement CPD programme for nursing educators; support emotional competence development; improve nurse education quality and emotional well-being.
3. Resource allocation for technology in emotional competence	Channel resources (time, money) for technology; more research needed on nurse educators’ needs; caution with immersive technologies, such as VR and Serious Games; situational analysis required.
4. Cautious adoption of immersive technologies	Use of serious games and VR requires caution; situational analysis needed; student proficiency and comfort issues; activity duration planning important.

## Conclusion

Emotional competence is recognised as vital for healthcare professionals, and innovative educational approaches are important for developing these skills in nursing students. The use of serious games promises significant potential which merits more research and investigation. These insights from the development and evaluation of a Serious Game for nursing students can inform more effective future educational initiatives. The paper also highlights the importance of considering the specific context of using such educational initiatives in different international and transnational programmes.

## Ethics and consent

The evaluation exercise did not entail a scientific research exercise. This paper reports the outcomes of an evaluation exercise rather than the results of a research study. Ethics clearance was therefore not required nor sought. Consent for participation from the participants was sought prior to their engagement in the evaluation exercise. Prior to allowing volunteering students to participate in the referred focus group discussions, the authors ensured that every participating student had sent an email to the indicated author (Daren Chircop) noting their acceptance to participate following the informative invitation email. The students’ individual reply emails assumed their individual consent to participate. The document in this link refers https://docs.google.com/document/d/1n4IQUTOUf6c48KcNIJ5HCAhp3izk_ih2/edit?usp=sharing&ouid=117399752553918572085&rtpof=true&sd=true.

## Data Availability

The datasets collected in the focus group discussions cannot be shared due to ethical and privacy concerns since the students who participated in the evaluation exercise being reported in this paper were participating in their capacity as volunteers to participate in the evaluation of the serious game as an educational tool. At no point were the participants advised that datasets from the group discussions will be made available through open access platforms and therefore consent in this specific regard is missing. The request for access to the data may be made directly to the authors of the paper mainly through email address maria.cassar@um.edu.mt. Access to data will however only be provided if and when the authors secure written consent by the participants authorising the authors to grant the respective requested access.
