# Supplementary material for: Serious game in developing emotional competence: An evaluation report
Source: Open Res Eur. 2026 Apr 9;5:354. Originally published 2025 Nov 20. [Version 2] doi: 10.12688/openreseurope.21634.2 (PMC13070205; doi:10.12688/openreseurope.21634.2)
Supplement: Peer review history

# Reviewer report: Reviewer response for version 2

**DOI:** 10.21956/openreseurope.25411.r72097

**Published:** 2026-04-20

**Authors:** Ioannis Zervas

**Affiliations:** 1 University of Macedonia, Thessaloniki, Greece

Thank you for revising the manuscript. The paper is now clearer in a number of important respects. In particular, it now provides more concrete information on the participants, the use of two focus groups, and the thematic analysis process, which makes the evaluation procedure easier to follow.

 I also appreciated the clearer explanation that the paper reports an educational evaluation exercise and not a formal research study. In addition, the revised text now separates participant responses more clearly from the authors’ interpretations and recommendations, which improves the overall presentation of the findings.

 Overall, I consider the manuscript suitable for approval.

Is the study design appropriate and does the work have academic merit?

Yes

Is the work clearly and accurately presented and does it cite the current literature?

Partly

If applicable, is the statistical analysis and its interpretation appropriate?

Not applicable

Are all the source data underlying the results available to ensure full reproducibility?

Partly

Are the conclusions drawn adequately supported by the results?

Partly

Are sufficient details of methods and analysis provided to allow replication by others?

Partly

Reviewer Expertise:

Education and training research, with experience in educational evaluation and digital learning approaches applied in professional contexts

I confirm that I have read this submission and believe that I have an appropriate level of expertise to confirm that it is of an acceptable scientific standard.

# Reviewer report: Reviewer response for version 2

**DOI:** 10.21956/openreseurope.25411.r72095

**Published:** 2026-04-11

**Authors:** Paula Diogo

**Affiliations:** 1 Nursing Research Innovation and Development Centre of Lisbon (CIDNUR), School of Nursing, University of Lisbon, Lisbon, Lisbon, Portugal

The suggested revisions have been addressed by the authors of the manuscript. I consider that it meets the requirements for **acceptance for indexing**.

Is the study design appropriate and does the work have academic merit?

Yes

Is the work clearly and accurately presented and does it cite the current literature?

Partly

If applicable, is the statistical analysis and its interpretation appropriate?

Not applicable

Are all the source data underlying the results available to ensure full reproducibility?

Yes

Are the conclusions drawn adequately supported by the results?

Yes

Are sufficient details of methods and analysis provided to allow replication by others?

Partly

Reviewer Expertise:

Emotional labour in nursing; Emotional competence; Emotional management in nursing practice

I confirm that I have read this submission and believe that I have an appropriate level of expertise to confirm that it is of an acceptable scientific standard.

# Response

**Published:** 2026-04-11

**Authors:** Maria Cassar

**Affiliations:** Nursing, University of Malta, Msida, Tal qroqq, Malta

Thanks for reviewing the revised submission. We welcome your approval of the paper. Maria Cassar

# Reviewer report: Reviewer response for version 1

**DOI:** 10.21956/openreseurope.23403.r65863

**Published:** 2026-01-05

**Authors:** Paula Diogo

**Affiliations:** 1 Nursing Research Innovation and Development Centre of Lisbon (CIDNUR), School of Nursing, University of Lisbon, Lisbon, Lisbon, Portugal

**REPORT**

 I would like to express my gratitude for the opportunity to review your manuscript.

 The present qualitative study focuses on the emotional competence of nursing students and evaluates the development and application of a Serious Game as an educational tool to promote this essential competence in the performance of nurses. Congratulations to the authors for tackling such an important issue.

 The following comments are provided with the aim of further enhancing the clarity, coherence, and overall presentation of the ideas discussed in the manuscript.

**- Abstract**

 It is hereby proposed that the methods section of the Abstract be revised to include additional information, such as the number of participants, the number of focus groups, and which author or framework was followed to perform the content analysis of the qualitative data.

**- Introduction**

 The Introduction section could be strengthened by clarifying two concepts. First, it would be beneficial to define emotional competence. Second, it would be prudent to clarify Serious Games.

 I also propose to include a rationale of Emotional Labor in Nursing from the perspective of emotional management in encounters between nurses and patients.  To strengthen the argument, I suggest two studies that highlights emotional labor in nursing and the emotional competence of nurses Ref no.1 & Ref no. 2

**- Methods**

 It is necessary to determine whether authorization from the ethics committee is required. It is hereby proposed that this matter be reviewed and validated by the institution's ethics committee.

 It is hereby proposed that the number of participants and other pertinent characteristics include. like age and year of study.

 To ensure a comprehensive understanding of the focus groups, additional information should be included, such as the number of focus groups held, the number of students who participated in each group, the way they were conducted, and other pertinent details.

Is the study design appropriate and does the work have academic merit?

Yes

Is the work clearly and accurately presented and does it cite the current literature?

Partly

If applicable, is the statistical analysis and its interpretation appropriate?

Not applicable

Are all the source data underlying the results available to ensure full reproducibility?

Yes

Are the conclusions drawn adequately supported by the results?

Yes

Are sufficient details of methods and analysis provided to allow replication by others?

Partly

Reviewer Expertise:

Emotional labour in nursing; Emotional competence; Emotional management in nursing practice

I confirm that I have read this submission and believe that I have an appropriate level of expertise to confirm that it is of an acceptable scientific standard, however I have significant reservations, as outlined above.

## References

[1] : Emotional labor of nurses in the front line against the COVID-19 pandemic. *Revista Brasileira de Enfermagem* .2021;74(suppl 1) : 10.1590/0034-7167-2020-0660 10.1590/0034-7167-2020-0660 33886837

[2] : Emotional Labor in Pediatric Palliative Care: A Scoping Review. *Nursing Reports* .2025;15(4) : 10.3390/nursrep15040118 10.3390/nursrep15040118 40333038 PMC12029664

# Response

**Published:** 2026-03-19

**Authors:** Maria Cassar

**Affiliations:** Nursing, University of Malta, Msida, Tal qroqq, Malta

Thank you for reviewing the paper and your constructive feedback and guidance. We hope that the changes we have completed in the revised submission address concerns sufficiently.

# Reviewer report: Reviewer response for version 1

**DOI:** 10.21956/openreseurope.23403.r65865

**Published:** 2025-12-30

**Authors:** Ana Žepina Puzić

**Affiliations:** 1 Šibenik University of Applied Sciences, Šibenik, Croatia

 This Brief Report presents an evaluation of a Serious Game designed to support the development of emotional competence among undergraduate nursing students. The game was implemented within a three-day workshop on emotional competence at the University of Malta, and its educational value was explored through audio-recorded focus group discussions with participating students. The evaluation adopts a qualitative approach, with thematic analysis used to identify key themes related to students’ motivation for participation, perceived benefits of the workshop and game, and suggestions for future initiatives. The topic is timely and relevant to nursing education, particularly given growing interest in immersive technologies and the development of non-technical skills such as emotional competence.

 clarity is occasionally reduced by: 1. conceptual overlap between *evaluation* and *qualitative research*, particularly in the Methods and Rigour sections;2.some repetition and overextension of the introduction relative to the Brief Report format;3. occasional ambiguity regarding the intended scope and transferability of the findings.

 While the overall methodological approach is described, several important details are missing or insufficiently elaborated to support replication or informed adaptation in other contexts, including: 1.the exact number of participants and focus groups conducted;2. key participant characteristics (e.g. year of study, prior exposure to VR);3.greater detail on how thematic analysis was operationalised in practice (e.g. coding process, development of themes, handling of divergent interpretations).In addition, the description of rigour (audit by co-authors, reflexive journals) resembles that of a qualitative research study, which again blurs the distinction between evaluation and research.

 No statistical analysis is reported in this evaluation, and therefore this criterion is not applicable.

 The conclusions regarding the perceived educational value of the Serious Game and workshop are generally supported by the participant excerpts and identified themes. However, some broader implications and recommendations (e.g. international hubs, universal emotional competence requirements, future-proofing education through AI) extend beyond what can reasonably be supported by a small-scale, context-specific evaluation.

Is the study design appropriate and does the work have academic merit?

Yes

Is the work clearly and accurately presented and does it cite the current literature?

Partly

If applicable, is the statistical analysis and its interpretation appropriate?

Not applicable

Are all the source data underlying the results available to ensure full reproducibility?

Partly

Are the conclusions drawn adequately supported by the results?

Partly

Are sufficient details of methods and analysis provided to allow replication by others?

Partly

Reviewer Expertise:

Based on my professional expertise in nursing and health professions education , I consider myself competent to assess its scientific quality.

I confirm that I have read this submission and believe that I have an appropriate level of expertise to confirm that it is of an acceptable scientific standard, however I have significant reservations, as outlined above.

# Response

**Published:** 2026-03-19

**Authors:** Maria Cassar

**Affiliations:** Nursing, University of Malta, Msida, Tal qroqq, Malta

Thank you very much for reviewing the paper and for your constructive comments. We have revised the paper following your guidance and believed it has been enhanced in clarity and detail.  We are unable to report the participants' previous exposure and experience with using VR because we are missing such info. Thanks again

# Reviewer report: Reviewer response for version 1

**DOI:** 10.21956/openreseurope.23403.r65868

**Published:** 2025-12-16

**Authors:** Ioannis Zervas

**Affiliations:** 1 University of Macedonia, Thessaloniki, Greece

**Summary**:

 The brief report presents an evaluation of a serious game designed to support the development of emotional competence among nursing students. The evaluation was conducted through a workshop setting, combining engagement with the game and subsequent focus group discussions. The aim of the exercise is clearly stated, and the authors provide a detailed description of both the game and the educational context in which it was used. As a reviewer-reader, I occasionally found it unclear how far the authors intend the evaluation findings to be taken, particularly in methodological terms.

**Scope and Positioning:**

 The manuscript explicitly frames the work as an evaluation exercise rather than a formal research study, which is appropriate for a Brief Report. This distinction helps set expectations for the reader. That said, in several places the language and analytical framing move closer to that of a qualitative research study, especially through references to thematic analysis and analytical rigour. Clarifying this boundary more consistently throughout the text would strengthen the positioning of the report and avoid potential confusion regarding its intended scope.

**Methodological Approach:**

 Using focus groups to capture students’ experiences is a reasonable choice for an educational evaluation of this kind. However, the description of the analytical process remains rather general. While the authors refer to the six steps of thematic analysis, it is not always clear how these steps were applied in practice or how the themes were agreed upon. A slightly more concrete account of the analytical process would help the reader better understand how the findings were derived, without necessarily turning the evaluation into a full research study. At present, this part of the report reads more as a summary of intent than as a description of analytical practice.

**Findings and Interpretation:**

 The three themes identified reflect issues that are clearly relevant to nursing education and emotional competence. The participants’ excerpts are informative and give a good sense of the workshop experience. At the same time, the discussion occasionally moves quickly from participant accounts to broader interpretations. A clearer distinction between what participants reported and how the authors interpret these accounts would improve the transparency of the findings and their educational relevance.

**Implications and Conclusions:**

 The conclusions highlight the potential of serious games in supporting emotional competence development, which aligns with the overall focus of the report. Some of the implications, however, appear to go beyond what can be supported by a small-scale, context-specific evaluation. Framing these implications more cautiously, and tying them more explicitly to the evaluation context, would strengthen the credibility of the conclusions and help readers understand where the contribution realistically sits.

**Overall Assessment:**

 Overall, the report addresses a relevant and timely topic and offers useful reflections for educators interested in innovative approaches to emotional competence development. The manuscript would benefit from clearer and more consistent positioning as an evaluation exercise, as well as from greater transparency in the description of the analytical process. With these clarifications, the report would sit more comfortably within the Brief Report format of Open Research Europe and better reflect its intended evaluative purpose.

Is the study design appropriate and does the work have academic merit?

Yes

Is the work clearly and accurately presented and does it cite the current literature?

Partly

If applicable, is the statistical analysis and its interpretation appropriate?

Not applicable

Are all the source data underlying the results available to ensure full reproducibility?

Partly

Are the conclusions drawn adequately supported by the results?

Partly

Are sufficient details of methods and analysis provided to allow replication by others?

Partly

Reviewer Expertise:

Education and training research, with experience in educational evaluation and digital learning approaches applied in professional contexts

I confirm that I have read this submission and believe that I have an appropriate level of expertise to confirm that it is of an acceptable scientific standard, however I have significant reservations, as outlined above.

# Response

**Published:** 2026-03-19

**Authors:** Maria Cassar

**Affiliations:** Nursing, University of Malta, Msida, Tal qroqq, Malta

Thank you so much for the detailed constructive feedback which you sent us. We are grateful for your review of our paper. We have sought to address all the guidance which you offered and are hopeful that the quality of the paper has been enhanced. Thanks again
